# A computational pharmacokinetic-pharmacodynamic framework for simulating and comparing individualized levodopa dosing in Parkinson’s disease

**DOI:** 10.3389/fphar.2026.1817435

**Published:** 2026-05-29

**Authors:** Sayaphat Suksai, Suthep Suantai, Pawarit Wandee

**Affiliations:** 1 Department of Mathematics, Faculty of Science, Srinakharinwirot University, Bangkok, Thailand; 2 Department of Mathematics, Faculty of Science, Chiang Mai University, Chiang Mai, Thailand

**Keywords:** computational simulation, individualized dosing, levodopa, Parkinson’s disease, pharmacokinetic–pharmacodynamic modeling, sensitivity analysis

## Abstract

Parkinson’s disease is associated with substantial inter-patient variability in motor response to levodopa therapy, making long-term dose selection and scheduling challenging. Computational modeling provides a systematic approach for investigating how pharmacokinetic and pharmacodynamic variability may influence treatment outcomes. This study presents a computational pharmacokinetic–pharmacodynamic framework for simulating and comparing individualized levodopa dosing strategies in Parkinson’s disease. The framework integrates multi-compartment drug disposition, effect-site dynamics, and a nonlinear exposure–response relationship, using parameter ranges derived from published clinical and modeling studies. A heterogeneous virtual cohort was generated to represent variability in absorption, clearance, distribution, and pharmacodynamic sensitivity, and simulation-based optimization was applied to evaluate the effects of dose size, dosing interval, and timing across representative patient scenarios. Simulations demonstrated how alternative dosing schedules influence effect-site exposure stability and predicted therapeutic response patterns under inter-patient variability. Sensitivity analysis further identified key parameters contributing most strongly to simulated exposure–response behavior. Rather than providing prescriptive clinical recommendations, the proposed framework serves as an *in silico* platform for comparing dosing regimens, evaluating scenario-specific strategies, and generating hypotheses for future data-driven validation. These findings highlight the potential of simulation-based PK–PD modeling to support individualized treatment exploration and future model-informed dosing research in Parkinson’s disease.

## Introduction

Parkinson’s disease (PD) is a progressive neurodegenerative disorder and the second most common condition of its kind after Alzheimer’s disease, affecting approximately 1%–2% of individuals over the age of 60 worldwide (Kalia and Lang, 2015; Contin and Martinelli, 2010). The hallmark pathology of PD arises from degeneration of dopaminergic neurons within the nigrostriatal pathway, leading to a substantial reduction in dopamine availability and consequent motor symptoms such as bradykinesia, rigidity, tremor, and postural instability (Contin and Martinelli, 2010). Broader biological and modeling perspectives have emphasized the value of quantitative approaches for understanding complex dynamical processes in disease progression (Dingemanse and Appel-Dingemanse, 2007). Early clinical manifestations—including tremor, gait abnormalities, and muscle stiffness—typically emerge near disease onset and often worsen with advancing pathology (Kalia and Lang, 2015; Contin and Martinelli, 2010).

Levodopa (L-dopa), a metabolic precursor of dopamine, remains the most effective pharmacological intervention for improving motor symptoms because it crosses the blood–brain barrier and restores synaptic dopamine levels (Westin et al., 2011; Senek et al., 2020). However, prolonged levodopa administration is frequently associated with the development of motor fluctuations and dyskinesia, which significantly impair functional independence and quality of life (Contin et al., 2001; Olanow and Obeso, 2011; Johansson et al., 2018; Senek et al., 2018). Treatment response varies widely among patients due to multiple pharmacokinetic and pharmacodynamic factors—including delayed gastric emptying, dietary influences, enzyme inhibition, and variability in drug absorption—as well as patient-specific determinants such as age, disease severity, body weight, and comorbidities (Senek et al., 2020; Senek et al., 2018; Nutt, 2008; Cao et al., 2024; Müller, 2013; Leta et al., 2023; Nyholm, 2006). In addition, gastrointestinal barriers such as impaired intestinal transit and altered levodopa absorption have been increasingly recognized as clinically important contributors to erratic response patterns in PD (Leta et al., 2023). These sources of heterogeneity complicate efforts to personalize levodopa therapy in clinical practice. Pharmacokinetic–pharmacodynamic (PK–PD) modeling provides a systematic framework for characterizing levodopa absorption, distribution, and motor response, enabling quantitative evaluation of treatment strategies under physiological and behavioral variability (Stocchi et al., 2008; Olanow et al., 2008; Espay et al., 2018; Troconiz et al., 1998; Jorga et al., 2000; Triggs et al., 1996; Mao et al., 2013; Parkinson Study Group, 2000; Mould and Upton, 2012). Prior modeling studies have contributed critical insights into oral, extended-release, and infusion-based formulations; however, many have focused on specific dosing regimens or narrowly defined clinical populations, limiting their ability to capture inter-individual variability and the competing demands of maximizing motor benefit while reducing adverse effects (Nutt, 2008; Stocchi et al., 2008; Troconiz et al., 1998; Jorga et al., 2000; Othman and Dutta, 2014). As a result, the development of integrative and transparent modeling systems remains essential for advancing individualized levodopa dosing.

In this study, we introduce a PK–PD modeling framework that incorporates patient variability, clinically relevant motor outcomes, and numerical optimization to evaluate individualized levodopa regimens. Using a virtual cohort with heterogeneous pharmacological and physiological profiles, the framework enables systematic comparison of dosing strategies and provides mechanistic insight into factors driving therapeutic variability. Ultimately, this work aims to support evidence-based and personalized treatment planning in Parkinson’s disease.

### Related PK-PD modeling studies and emerging directions

Previous PK–PD studies of levodopa therapy have provided important insights into population-based approaches, formulation-specific models, and the quantitative assessment of motor complications (Nutt, 2008; Stocchi et al., 2008; Olanow et al., 2008; Espay et al., 2018; Troconiz et al., 1998; Jorga et al., 2000; Triggs et al., 1996; Mao et al., 2013; Othman and Dutta, 2014). These studies have improved understanding of variability in levodopa pharmacokinetics and have supported comparative evaluation of oral and infusion-based regimens (Westin et al., 2011; Senek et al., 2020; Othman and Dutta, 2014; Schröter et al., 2022). More recent modeling efforts have increasingly incorporated patient covariates, individualized motor-response patterns, and disease-progression elements to better capture heterogeneity in treatment outcomes (Senek et al., 2020; Jorga et al., 2000; Triggs et al., 1996; Mao et al., 2013; Parkinson Study Group, 2000; Mould and Upton, 2012). Beyond formulation-specific PK–PD analyses, recent translational developments have also highlighted the emerging role of digital biomarkers, wearable monitoring systems, and technology-assisted symptom assessment in Parkinson’s disease management (Sun et al., 2024; Bougea, 2024; Rodríguez-Martín and Pérez-López, 2024). Collectively, these developments suggest that quantitative PK–PD frameworks may serve not only as tools for comparing dosing regimens, but also as computational platforms for integrating multimodal patient information and supporting future model-informed precision dosing strategies (Sun et al., 2024; Minichmayr et al., 2024; Kumar et al., 2018).

### Study objectives and conceptual framework

**FIGURE 1:**
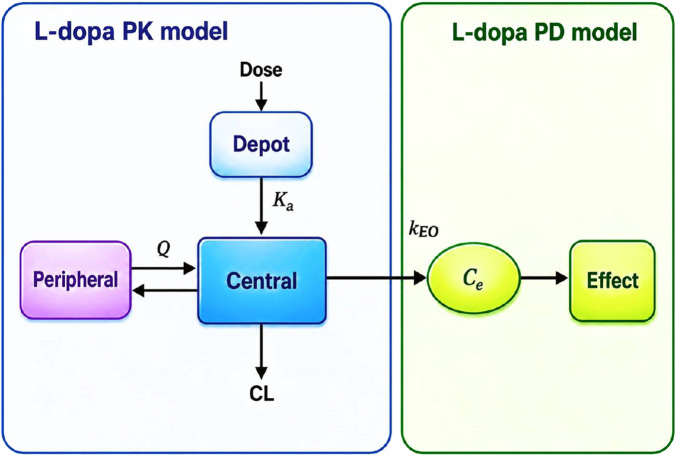
Schematic representation of the pharmacokinetic–pharmacodynamic (PK–PD) model of levodopa, illustrating drug absorption from the gastrointestinal tract, distribution through plasma and peripheral compartments, and delivery to the brain effect site. The model captures the bidirectional relationships between pharmacokinetic processes and pharmacodynamic responses, forming the mechanistic basis for individualized dose optimization in Parkinson’s disease.

This work introduces an integrative PK–PD modeling framework to investigate individualized levodopa dosing strategies by combining published pharmacological data, variability in patient characteristics, and numerical optimization (Nutt, 2008; Stocchi et al., 2008; Olanow et al., 2008; Espay et al., 2018; Troconiz et al., 1998; Jorga et al., 2000; Triggs et al., 1996; Mao et al., 2013; Othman and Dutta, 2014). Rather than focusing solely on standard dosing schedules, the framework allows systematic evaluation of oral, fractionated, and infusion-based regimens across diverse simulated patient profiles (Westin et al., 2011; Senek et al., 2020; Othman and Dutta, 2014; Schröter et al., 2022). In addition, concepts related to technology-assisted or microtablet-based personalized dosing can be explored within this structure (Johansson et al., 2018; Simon et al., 2016; Holford et al., 2006). The overarching goal is to provide a practical and mechanistically interpretable computational platform for comparing dosing strategies under inter-patient variability and for informing future personalized therapeutic decision-support research in PD, rather than serving as a direct bedside clinical decision tool (Minichmayr et al., 2024; Kumar et al., 2018). The conceptual structure of the proposed framework is illustrated in Figure 1, which summarizes the gastrointestinal absorption pathway, the central and peripheral pharmacokinetic compartments, and the effect-site linkage used to represent delayed pharmacodynamic response and individualized dose optimization.

### PK-PD model structure and optimization framework

To investigate individualized levodopa therapy, this study employs a pharmacokinetic–pharmacodynamic (PK–PD) model that characterizes levodopa absorption, distribution, and clinical effect across key physiological components relevant to oral administration (Figure 1). The pharmacokinetic structure consists of a gastrointestinal depot linked to a central plasma compartment, with bidirectional exchange with a peripheral compartment. This structure is consistent with established levodopa PK and PK–PD models and provides a mechanistically interpretable basis for regimen comparison (Westin et al., 2011; Troconiz et al., 1998; Jorga et al., 2000; Triggs et al., 1996; Mao et al., 2013; Othman and Dutta, 2014). An effect-site compartment is incorporated to represent the temporal dissociation between plasma levodopa concentrations and observed motor response, a feature that is essential for capturing the hysteresis commonly seen in Parkinson’s disease and in prior levodopa response studies (Nutt, 2008; Troconiz et al., 1998; Mao et al., 2013). This model structure provides a mechanistic framework for quantifying dose–response relationships and evaluating how physiological variability may influence therapeutic outcomes.

**FIGURE 2:**
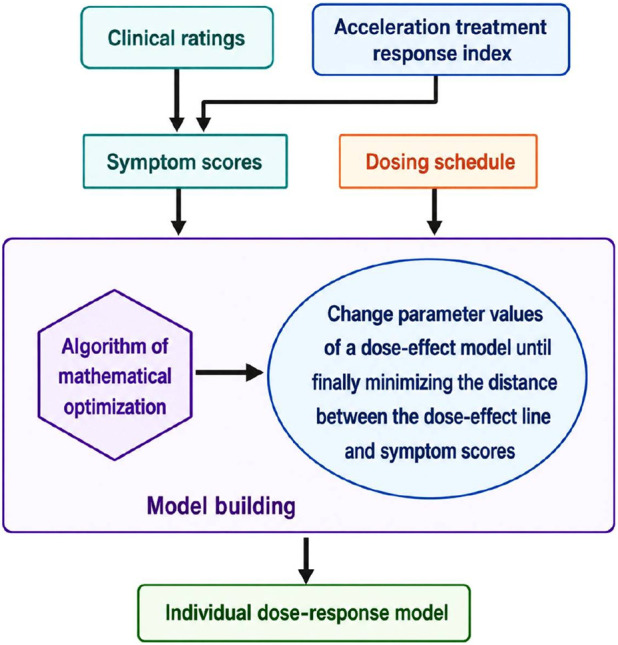
Clinical ratings and symptom scores are iteratively integrated with dosing schedules and optimization algorithms to refine model parameters until the simulated dose–response curve converges with predefined reference response profiles. This individualized PK–PD framework enables evaluation and comparison of alternative levodopa treatment strategies for Parkinson’s disease management.

The optimization-oriented workflow of the proposed framework is summarized in Figure 2. Model parameters were informed by published pharmacological and clinical data reflecting typical characteristics of individuals with Parkinson’s disease and literature-reported levodopa treatment contexts (Westin et al., 2011; Senek et al., 2020; Troconiz et al., 1998; Jorga et al., 2000; Triggs et al., 1996; Mao et al., 2013; Othman and Dutta, 2014). To enable exploration of optimized dosing regimens, the PK–PD model was integrated with a numerical optimization procedure that evaluates alternative dosing schedules—including dose magnitude, frequency, and timing—with the objective of improving motor control while reducing the risk of motor fluctuations and dyskinesia (Figure 2) in a comparative *in silico* setting (Contin et al., 2001; Olanow and Obeso, 2011; Johansson et al., 2018; Senek et al., 2018; Stocchi et al., 2008; Olanow et al., 2008; Espay et al., 2018). Rather than functioning as a real-time closed-loop system, the proposed approach serves as an *in silico* platform for comparing oral and infusion-based strategies under inter-patient variability and for generating mechanistic hypotheses to support future personalized dosing studies.

By leveraging such models, this study aims to provide a quantitative basis for exploring individualized levodopa dosing strategies in Parkinson’s disease. The proposed PK–PD framework enables systematic evaluation of dose–response relationships under heterogeneous patient conditions and highlights factors that may contribute to motor fluctuations and dyskinesia. Because both motor and non-motor fluctuations influence patients’ quality of life, they must be considered when interpreting model-based dosing outcomes. In this broader translational context, the framework may also provide a useful foundation for future integration with digital monitoring technologies and symptom-tracking platforms designed to capture patient-specific response variability in real-world settings (Schröter et al., 2022; Chan et al., 2005; Contin et al., 2022).

In this framework, levodopa exposure is linked to clinical motor outcomes through a standardized response measure consistent with prior PK–PD and monitoring studies (Mao et al., 2013; Holford et al., 2006). Several key covariates known to influence levodopa kinetics are incorporated, including body weight, disease stage, and gastrointestinal function. Variations in body weight can modify distribution-related parameters, whereas delayed gastric emptying and reduced gastrointestinal motility—commonly observed in advanced PD—may lead to variable absorption and fluctuations in therapeutic response. Accounting for such physiological sources of variability is therefore essential when evaluating individualized dosing strategies. Recent evidence further indicates that gastrointestinal barriers to levodopa transport and absorption may substantially influence exposure variability and contribute to inconsistent therapeutic response, particularly in patients with advanced or fluctuating disease (Leta et al., 2023; Nyholm, 2006).

### PK-PD model formulation

The pharmacokinetic–pharmacodynamic (PK–PD) model is formulated using ordinary differential equations to describe levodopa absorption, distribution, and elimination. Consistent with widely used levodopa population PK and PK–PD frameworks, the PK component includes a gastrointestinal depot linked to a central compartment, with distribution to peripheral tissues (Figure 1) (Westin et al., 2011; Senek et al., 2020; Johansson et al., 2018; Senek et al., 2018; Nutt, 2008; Cao et al., 2024; Müller, 2013; Leta et al., 2023; Nyholm, 2006; Stocchi et al., 2008; Bougea, 2024; Rodríguez-Martín and Pérez-López, 2024; Minichmayr et al., 2024). To represent the temporal dissociation between systemic exposure and clinical motor response, a biophase (effect-site) compartment is incorporated. This effect-site model provides a simplified functional representation of drug delivery to the neural structures associated with motor effects, rather than a detailed anatomical description of brain disposition.

### Oral absorption

For oral administration, levodopa enters the system through a first-order absorption process from the gastrointestinal depot,

$$\frac{dA_{gut}}{dt}=-k_{a}A_{gut},$$

where $A_{gut}$ is the amount of levodopa in the gastrointestinal tract (mg) and $k_{a}$ is the first-order absorption rate constant (*h* ^*-1*^). This component captures the sensitivity of systemic exposure to gastrointestinal function, which is clinically relevant in advanced PD. In particular, delayed gastric emptying and gastrointestinal dysmotility may alter the rate and extent of levodopa absorption, thereby contributing to response variability across patients.

### Infusion input

For continuous infusion, drug input to the central compartment is represented as a zero-order rate,

$$R_{in}=F\cdot F_{\mathrm{int}},$$

where $F_{\mathrm{int}}$ is the infusion rate (mg/min) and F is the bioavailability factor. This input term is incorporated into the central compartment balance rather than treated as a standalone differential equation.

### Distribution between central and peripheral compartments

The exchange between the central (plasma) and peripheral compartments is defined using an inter-compartmental clearance approach,

$$\frac{dA_{peripheral}}{dt}=\frac{CL_{\mathrm{int}}}{V_{plasma}}\cdot A_{plasma}-\frac{CL_{\mathrm{int}}}{V_{peripheral}}\cdot A_{peripheral},$$ (1)

where $A_{plasma}$ and $A_{peripheral}$ denote levodopa amounts (mg), $CL_{\mathrm{int}}$ is the inter-compartmental clearance (L/min), and $V_{plasma}$ and $V_{peripheral}$ are compartment volumes (L). This formulation captures bidirectional distribution governed by concentration gradients.

### Biophase (effect-site) compartment

To link systemic exposure to the delayed motor response, we introduce a biophase (effect-site) compartment receiving drug from the systemic distribution pathway,

$$\frac{dA_{effect}}{dt}=\frac{CL_{\mathrm{int}}}{V_{peripheral}}\cdot A_{peripheral}-\frac{CL_{\mathrm{int}}}{V_{effect}}\cdot A_{effect},$$ (2)

where $A_{effect}$ is the levodopa amount in the biophase (effect-site) compartment and $V_{effect}$ is its apparent volume. This component is intended to represent functional exposure relevant to motor effects in a parsimonious manner. Accordingly, the effect-site compartment should be interpreted as a phenomenological bridge between systemic pharmacokinetics and delayed pharmacodynamic response, rather than as a literal anatomical compartment.

### Elimination

Systemic elimination from the central compartment is modeled as a first-order process with clearance CL . The elimination term is given by

$$\frac{dA_{plasma}}{dt}=-CL\cdot \frac{A_{plasma}}{V_{plasma}},$$ (3)

where CL is the total clearance (*L/min*). For oral and infusion regimens, this elimination term is combined with the corresponding input and distribution terms in the full central compartment mass balance.

### Effect-site dynamics and pharmacodynamic response

The therapeutic response is assumed to depend on the effect-site concentration $C_{e}$ . To capture the temporal delay between effect-site exposure and clinical effect, we use an equilibration model

$$\frac{dC_{e}}{dt}=k_{e}\cdot \left(\frac{A_{effect}}{V_{effect}}-C_{e}\right),$$ (4)

where $k_{e}$ is the effect-site equilibration rate constant. The motor response is described by a sigmoidal $E_{\max}$ relationship,

$$E=E_{0}+\frac{\text{Emax}\cdot C_{e}^{\gamma }}{C_{50}^{\gamma }+C_{e}^{\gamma }}.$$ (5)

where $E_{0}$ is baseline motor status, $E_{\max}$ is the maximum achievable effect, $C_{50}$ is the effect-site concentration producing 50% of $E_{\max}$ , and γ is the Hill coefficient.

Together, these components define a coherent PK-PD framework for describing levodopa exposure and the associated motor response. The model is represented by a system of Equations 1–5 capturing the key processes of dosing input, absorption, distribution, elimination, and effect-site response. In particular, Equation 5 links $C_{e}$ to the modeled therapeutic effect through a sigmoidal $E_{\max}$ relationship, thereby providing a parsimonious representation of levodopa’s concentration-effect profile. To account for inter-individual variability, targeted sensitivity analyses were used to identify parameters exerting the greatest influence on model outputs. Based on these analyses, a subset of primary parameters was adjusted across simulated patient profiles, whereas the remaining parameters were fixed to representative literature-informed values (Jorga et al., 2000; Triggs et al., 1996; Contin et al., 2022). This strategy was adopted to balance individualized adaptation, parameter identifiability, and consistency with prior levodopa PK-PD studies.

We then incorporated a numerical optimization procedure to refine dosing-related variables by minimizing the discrepancy between the model-predicted response and a predefined reference response profile derived from the TRS-based motor outcome framework (Mao et al., 2013; Holford et al., 2006). The objective function was defined as

$$\text{Objective Function}=\sum_{i=1}^{N}{\left(E_{ref,i}-E_{pred,i}\right)}^{2},$$ (6)

where $E_{ref,i}$ denotes the reference therapeutic response at time point i , $E_{pred,i}$ is the corresponding model prediction, and N is the number of evaluation time points. By minimizing the sum of squared residuals, the optimization procedure explores dose size, dosing interval, and timing to identify regimens that improve simulated motor control while reducing excessive fluctuations associated with OFF periods and dyskinesia. This optimization step is intended to support comparative *in silico* evaluation of alternative regimens rather than to provide patient-specific clinical recommendations in its current form.

To evaluate the proposed framework, simulations were conducted using 100 virtual patient profiles generated from population-based parameter sampling. Each virtual profile incorporated variability in key PK and PD parameters, including age, body weight, disease stage, and baseline dopaminergic tone. The simulations characterized concentration-time behavior across multiple dosing cycles and compared alternative dosing strategies under inter-patient variability. Optimized regimens consisted of loading and maintenance components applied within the simulated environment.

**FIGURE 3:**
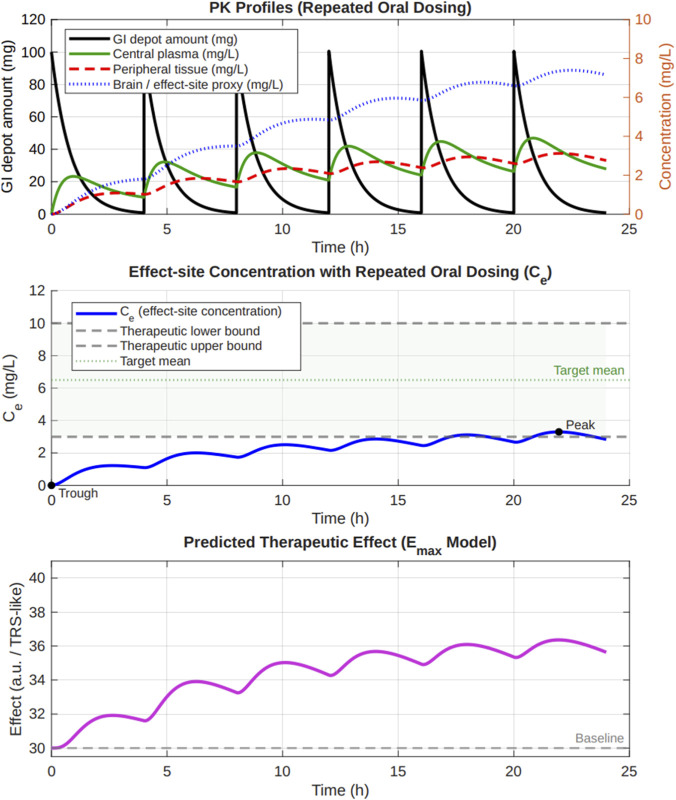
Simulated PK–PD profiles of levodopa during repeated oral dosing. Top: compartmental pharmacokinetic profiles of the gastrointestinal depot, central plasma, peripheral tissue, and brain/effect-site proxy. Middle: effect-site concentration $\left(C_{e}\right)$ with a shaded therapeutic reference window, lower and upper bounds, and peak–trough annotations illustrating exposure variability across dosing intervals. Bottom: predicted therapeutic effect based on a sigmoidal $E_{\max}$ model (TRS-like scale), showing progressive stabilization of the response trajectory under repeated dosing.

Figure 3 presents representative concentration-time trajectories across model compartments and illustrates how the optimization procedure can reduce variability in effect-site exposure across dosing intervals. Sensitivity analyses further identified parameters exerting the strongest influence on simulated outcomes, thereby supporting mechanistic interpretation of inter-patient heterogeneity within the framework. Although these findings support the plausibility and usefulness of the framework as an exploratory *in silico* tool, they should not be interpreted as independent external clinical validation. Overall, the results indicate that the model provides a structured platform for investigating individualized levodopa dosing strategies and for generating hypotheses for future data-rich or prospective validation studies.

## Methods

### Literature-based parameter selection

This study employed a pharmacokinetic-pharmacodynamic (PK-PD) modeling framework to simulate levodopa exposure and its associated motor response in Parkinson’s disease. Parameter inputs were obtained exclusively from peer-reviewed publications reporting clinically relevant levodopa regimens, quantitative PK/PK-PD analyses, and population-based parameter estimates. No individual patient-level data were used; all simulations were conducted entirely *in silico*.

Literature sources were selected based on their reporting of key pharmacokinetic descriptors, including absorption rate constants, clearance, distribution volumes, inter-individual variability terms, and covariate effects, as well as pharmacodynamic parameters related to motor response. Studies that provided information on motor fluctuations, dyskinesia, or formulations relevant to oral and infusion-based levodopa therapy were prioritized (Westin et al., 2011; Senek et al., 2020; Troconiz et al., 1998; Jorga et al., 2000; Triggs et al., 1996; Mao et al., 2013; Othman and Dutta, 2014; Chan et al., 2005; Othman et al., 2015; Contin et al., 2022). In addition, studies discussing gastrointestinal influences on levodopa absorption and variability in therapeutic response were considered when relevant to model interpretation and parameter plausibility (Leta et al., 2023; Nyholm, 2006).

Publications were excluded when essential structural model components, dosing details, or parameter estimates required for implementation were incomplete or inconsistent with established levodopa population PK/PK-PD frameworks. To ensure coherence with prior modeling literature, data extraction focused on parameters commonly influenced by gastrointestinal absorption, disease severity, body weight, and treatment regimen characteristics such as oral administration, continuous infusion, and related dosing contexts (Westin et al., 2011; Senek et al., 2020; Nutt, 2008; Müller, 2013; Othman and Dutta, 2014). This selection strategy enabled construction of a transparent, literature-informed variability structure suitable for reproducible *in silico* evaluation of alternative dosing strategies, while acknowledging that the present study was not intended as a formal systematic review.

### Model development

The PK-PD model was implemented in MATLAB. The pharmacokinetic component describes oral levodopa input via a gastrointestinal depot, followed by distribution between central and peripheral compartments and systemic elimination, consistent with established population PK/PK-PD models (Westin et al., 2011; Senek et al., 2020; Nutt, 2008; Troconiz et al., 1998; Jorga et al., 2000; Triggs et al., 1996; Mao et al., 2013; Othman and Dutta, 2014; Chan et al., 2005; Othman et al., 2015; Contin et al., 2022). To account for the temporal dissociation between plasma levodopa concentrations and clinical motor response, an effect-site (biophase) compartment was incorporated and linked to a sigmoidal $E_{\max}$ function governing the pharmacodynamic response (Nutt, 2008; Troconiz et al., 1998; Jorga et al., 2000; Triggs et al., 1996; Mao et al., 2013). Model parameters were informed by published levodopa PK/PK-PD studies and adjusted within physiologically plausible ranges to reflect inter-individual variability reported in the literature (Westin et al., 2011; Senek et al., 2020; Troconiz et al., 1998; Jorga et al., 2000; Triggs et al., 1996; Mao et al., 2013; Othman and Dutta, 2014; Chan et al., 2005; Othman et al., 2015; Contin et al., 2022). The model structure was designed to support comparison of alternative dosing strategies within a fully simulated (*in silico*) environment, prioritizing mechanistic interpretability over detailed anatomical representation of brain drug disposition. Accordingly, the framework was developed as an exploratory computational platform for regimen comparison and hypothesis generation, rather than as a validated patient-specific clinical prediction tool (Minichmayr et al., 2024; Kumar et al., 2018).

For the repeated oral-dosing simulations and local sensitivity analysis, a baseline parameter configuration was defined from literature-informed values and used as the nominal reference setting for computational experiments. In this configuration, the regimen consisted of 100 mg per dose administered every 4 h for six doses over a 24-h simulation horizon. The baseline PK-PD parameters were $k_{a}=1.2\;h^{-1}$ , $k_{12}=0.6\;h^{-1}$ , $k_{21}=0.5\;h^{-1}$ , $k_{13}=0.25\;h^{-1}$ , $k_{31}=0.20\;h^{-1}$ , $k_{e}=0.35\;h^{-1}$ , and $k_{e0}=0.6\;h^{-1}$ . The corresponding volume and PD parameters were $V_{p}=20\;L$ , $V_{per}=25\;L$ , $V_{b}=10\;L$ , $E_{0}=30$ , $E_{\max}=10$ , $EC_{50}=2.5\text{ mg}/L$ , and γ=2.0 . These values were used as the nominal baseline for repeated-dose simulation, while selected parameters were perturbed by ±20% in the local sensitivity analysis.

### Simulation design

Simulations were conducted for 100 virtual patient profiles to capture inter-individual variability. Patient characteristics included heterogeneity in age (50–80 years), body weight (50–80 kg), and disease severity (Hoehn–Yahr stages II–IV). The simulated cohort included both relatively stable responders and profiles exhibiting motor fluctuations, reflecting patterns commonly reported in advanced Parkinson’s disease (Contin et al., 2001; Johansson et al., 2018; Senek et al., 2018; Stocchi et al., 2008). Simulations were performed over a 24-h repeated-dosing horizon to evaluate exposure and response behavior under steady-state-like periodic dosing conditions. Individual PK-PD parameters were sampled independently from literature-informed parameter ranges derived from published levodopa PK/PK-PD analyses. This strategy enabled both population-level and patient-specific simulations under consistent assumptions regarding absorption variability, distribution kinetics, and exposure-response relationships (Westin et al., 2011; Senek et al., 2020; Troconiz et al., 1998; Jorga et al., 2000; Triggs et al., 1996; Mao et al., 2013; Othman and Dutta, 2014; Chan et al., 2005; Othman et al., 2015; Contin et al., 2022). A full covariance structure was not explicitly modeled in the present study. The resulting virtual cohort provided a quantitative basis for evaluating dosing strategies under inter-patient variability and for examining how differences in patient characteristics influence simulated therapeutic outcomes. Because the virtual cohort was literature-informed rather than derived from prospectively observed patient-level data, the simulated outputs should be interpreted as exploratory scenario-based results rather than direct clinical forecasts.

### Optimization strategy

To explore individualized dosing regimens, we defined a least-squares objective function to minimize the deviation between model-predicted responses and a predefined reference motor response profile informed by the TRS-based outcome framework (Mao et al., 2013; Holford et al., 2006). Dosing-related variables, including dose size, dosing interval, and timing, were iteratively adjusted within the simulated environment to improve motor response while limiting excessive fluctuations that may be associated with OFF periods and dyskinesia (Contin et al., 2001; Johansson et al., 2018; Senek et al., 2018; Stocchi et al., 2008; Olanow et al., 2008; Espay et al., 2018). A scenario-based iterative search procedure was used to compare alternative dosing regimens within predefined clinically plausible ranges of dose size, dosing interval, and timing. The optimization objective is defined in Equation 6, while the loading-dose and maintenance-dose formulations are given in Equations 7, 8, respectively. The optimization procedure considered both loading and maintenance components to evaluate alternative strategies across different virtual patient profiles. Loading doses were defined as initial doses intended to approach the target exposure range, whereas maintenance doses were used to sustain exposure over subsequent dosing intervals. Regimens were compared according to their ability to reduce peak-trough fluctuations, maintain effect-site exposure within the therapeutic reference window, and minimize deviation from the predefined reference response profile.

Targeted sensitivity analyses were performed to identify parameters with the greatest influence on model outputs. Results from this analysis guided which parameters were individualized across patient profiles, while remaining parameters were fixed to representative values informed by the literature to support identifiability and consistent comparisons across regimens (Jorga et al., 2000; Triggs et al., 1996; Contin et al., 2022; Brendel et al., 2007). In this way, the optimization framework was used to compare alternative regimens under controlled simulation assumptions rather than to infer uniquely optimal doses for real patients.

### Statistical analysis and validation

The predictive behavior of the proposed PK-PD model was assessed by comparing simulated outputs with reference trends reported in published population-level studies of levodopa-treated patients with Parkinson’s disease. This evaluation focused on whether the simulated exposure-response patterns were qualitatively and quantitatively consistent with reported clinical observations of motor improvement, motor fluctuations, and dyskinesia risk under different dosing regimens. Model-predicted Treatment Response Scale (TRS)-based profiles were compared with literature-derived reference response patterns to assess the model’s ability to reproduce clinically plausible therapeutic trends. Where appropriate, descriptive correlation and goodness-of-fit metrics were used to summarize the agreement between simulated and reference TRS trajectories. However, these analyses were intended to provide supportive rather than confirmatory evidence of model plausibility within the constraints of literature-informed parameterization. The present evaluation should therefore be interpreted as a literature-based plausibility assessment, not as formal external validation against an independent patient-level dataset (Minichmayr et al., 2024; Kumar et al., 2018).

Sensitivity analyses were conducted to identify parameters contributing most to variability in simulated outcomes, including the absorption rate constant ( $k_{a}$ ), systemic clearance ( CL ), and inter-compartmental clearance ( $CL_{\mathrm{int}}$ ). Parameters showing the greatest influence on TRS-related fluctuations were prioritized for individualized adjustment during optimization to support evaluation across heterogeneous virtual patient profiles. This sensitivity-guided strategy was intended to improve interpretability of model behavior and to highlight parameters most relevant for future individualized estimation and data collection. Simulations of alternative dosing strategies, including twice-daily, thrice-daily, and continuous infusion regimens, were used to compare concentration-time behavior and TRS-based response patterns in the simulated (*in silico*) setting. The optimization results suggest that the proposed framework can reduce deviations from target response profiles and limit excessive exposure fluctuations across dosing intervals. Overall, these analyses support the use of the model as an *in silico* exploratory platform for investigating individualized levodopa dosing strategies and for generating hypotheses for future data-rich or prospective validation studies, rather than for direct clinical decision-making in its current form.

### Population variability and virtual cohort generation

To account for inter-patient variability, 100 virtual patient profiles were generated by independent sampling from literature-informed PK/PK-PD parameter ranges. Key parameters—including clearance (CL), apparent volume of distribution ( $V_{d}$ ), absorption-related parameters, and PD potency terms such as $EC_{50}$ —were sampled within ranges reported in clinical populations [(Westin et al., 2011), (Senek et al., 2020), (Senek et al., 2018), (Senek et al., 2018; Troconiz et al., 1998; Jorga et al., 2000; Triggs et al., 1996; Mao et al., 2013), (Senek et al., 2018; Troconiz et al., 1998; Jorga et al., 2000; Triggs et al., 1996; Mao et al., 2013), (Chan et al., 2005; Othman et al., 2015; Contin et al., 2022), (Othman et al., 2017)]. A full covariance structure was not explicitly modeled in the present study. The resulting virtual cohort captured heterogeneity in age, disease stage, body weight, and dopaminergic sensitivity. By embedding this variability, the model enabled systematic comparison of dosing strategies across diverse simulated patient profiles and supported exploration of individualized parameter adjustment within the *in silico* optimization framework. Because these profiles were generated computationally from literature-informed parameter ranges rather than observed prospectively in individual patients, the resulting simulations should be interpreted as scenario-based evaluations rather than real-world forecasts. In future extensions, incorporation of richer symptom descriptors, including digitally derived non-motor markers, may further improve individualized characterization within such virtual cohort frameworks (Janssen Daalen et al., 2024).

### Oral dosage regimen of L-Dopa

Oral levodopa remains the most widely used regimen in Parkinson’s disease, but maintaining stable therapeutic exposure can be challenging due to variability in gastrointestinal absorption, delayed gastric emptying, dietary interactions, and inter-patient differences in clearance and distribution [(Nutt, 2008), (Müller, 2013; Leta et al., 2023; Nyholm, 2006), (Gupta et al., 2019)]. These factors motivate the evaluation of loading and maintenance dosing components within an *in silico* PK-PD framework. In the simulated setting, the loading dose was defined to approximate the amount required to achieve a reference target concentration based on the apparent volume of distribution,

$$\text{Loading Dose}=C_{\text{target}}\cdot V_{d},$$ (7)

where $C_{\text{target}}$ is the desired reference concentration and $V_{d}$ ​ is the apparent volume of distribution. The maintenance dose was then defined to replenish drug levels over a dosing interval τ

$$\text{Maintenance Dose}=CL\cdot C_{\text{target}}\cdot \tau ,$$ (8)

where CL is the systemic clearance and τ is the dosing interval. Units were handled consistently to ensure mass balance in the simulated calculations. These formulations provided a practical, simulation-based basis for exploring dose size and dosing frequency in the virtual cohort and for comparing alternative oral dosing strategies under inter-patient variability.

### Simulation insights

Simulations of alternative oral dosing regimens were conducted to explore how dose size and dosing interval influence exposure–response patterns across heterogeneous virtual patient profiles. The resulting concentration–time trajectories exhibited peaks and troughs characteristic of oral levodopa administration. Within the simulated setting, individualized parameter adjustments and optimization reduced deviations from target response profiles and limited excessive exposure fluctuations across dosing intervals. These findings support the potential utility of the proposed PK–PD framework as an *in silico* tool for comparing dosing strategies and generating hypotheses for future data-rich validation studies, particularly for patients with advanced disease or fluctuating responses (Sun et al., 2024; Bougea, 2024; Rodríguez-Martín and Pérez-López, 2024).

### Visualization of PK–PD profiles

Figure 3 presents model-simulated concentration–time curves and corresponding therapeutic response profiles. The top-left panel shows simulated pharmacokinetic behavior of levodopa across model compartments (gastrointestinal depot, plasma, peripheral tissues, and brain), illustrating temporal patterns of absorption, distribution, and elimination following dosing. The top-right panel displays brain concentration profiles under repeated dosing, highlighting how the optimization routine adjusts dose size and timing to reduce exposure fluctuations relative to predefined target ranges in the simulated setting. The bottom panel illustrates the associated therapeutic effect trajectory, starting from baseline, increasing toward a peak response, and declining in parallel with changes in brain exposure.

**FIGURE 4:**
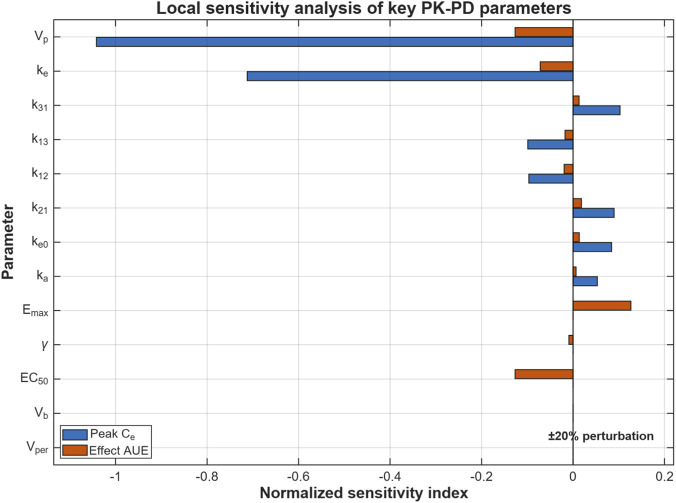
Local sensitivity analysis of key PK–PD parameters under repeated oral dosing. Each parameter was perturbed by ± 20% around its baseline value, and normalized sensitivity indices were calculated for peak effect-site concentration $\left(C_{e}\right)$ and therapeutic effect exposure. The results identify parameters with the greatest influence on simulated exposure–response behavior.

A local sensitivity analysis was performed to quantify the influence of key pharmacokinetic and pharmacodynamic parameters on simulated outcomes. As shown in Figure 4, parameters related to drug distribution and elimination (e.g., $V_{p}$ , $k_{e}$ ) exhibit strong influence on peak effect-site concentration, while pharmacodynamic parameters such as $EC_{50}$ and $E_{\max}$ primarily affect therapeutic response. Among the examined parameters, $V_{p}$ and $k_{e}$ show the largest negative sensitivity indices for peak effect-site concentration, indicating a strong influence on exposure dynamics, whereas $EC_{50}$ and $E_{\max}$ exert the clearest influence on therapeutic effect exposure. These findings support prioritization of key parameters for individualized model adjustment.

### Summary

Taken together, Tables 1, 2 and Figure 3 summarize the literature-informed parameterization and simulated PK–PD behavior of the proposed framework. The results indicate that incorporating inter-patient variability and optimization enables systematic exploration of dosing scenarios that reduce exposure oscillations and support therapeutic response patterns consistent with predefined target profiles. Overall, these findings support the utility of the framework as an *in silico* tool for comparing alternative regimens and guiding future validation studies toward individualized levodopa dosing.

**TABLE 1:** Demographic and clinical characteristics of Parkinson’s disease populations reported in studies used to inform model parameterization and virtual cohort design.

Author	Sample size, N (M/F)	Age (years)	Dose (mg/day)	Formulation/Route	Key information used	Model type
Triggs et al. (1996)	46 (31/15)	34–78	200–1,200	Oral	PK–PD parameters, inter-individual variability	PK–PD
Troconiz et al. (1998)	18	44–74	NR	Oral + entacapone	Population PD model, exposure–response	PD
Chan et al. (2005)	20 (12/8)	40–75	427–579	IV infusion	Within-subject PK variability	PK
Contin et al. (2001)	25 (13/12)	45–75	64–256	IV infusion	Kinetic–dynamic response	PK–PD
Westin et al. (2011)	27 (21/6)	48–81	816–2054	Duodenal infusion	PK–PD under infusion delivery	PK–PD
Mao et al. (2013)	30 (18/12)	51–78	200–550	Oral ER/IR	Extended-release PK–PD	PK–PD
Senek et al. (2018)	19 (11/8)	45–75	100–250	Microtablets	Population PK	PK
Senek et al. (2020)	68 (42/26)	NR	NR	LCIG	Intestinal gel population PK (infusion)	PK
Othman and Dutta, (2014)	38	NR	NR	Oral vs. LCIG	PK comparison, variability	PK
Othman et al. (2015)	24	NR	NR	LCIG	PK and variability	PK

Clinical review articles were used exclusively to describe disease background and clinical context in the Introduction and were not included in this table or used for PK/PD, parameter estimation; LCIG, levodopa–carbidopa intestinal gel; LC-oral, oral levodopa–carbidopa; NR, not reported; M/F, male/female; PK, pharmacokinetic; PD, pharmacodynamic; PK-PD, combined pharmacokinetic-pharmacodynamic.

**TABLE 2:** Pharmacodynamic parameters of levodopa therapy reported in selected PD and PK-PD modeling studies, including baseline effect ( $E_{0}$ ), maximum effect ( $E_{\max}$ ), potency parameter ( $C_{50}$ or $EC_{50}$ ), and Hill coefficient ( γ ), summarized to support literature-informed parameter ranges for virtual cohort simulations.

Study	γ	$E_{0}$	$E_{\max}$	Potency parameter
Nutt, (2008)	NR	NR	9.63	$C_{50}$ : 0.50
Contin et al. (2001)	NR	137, 146	1.06, 0.91, 1.30, 2.24	$C_{50}$ : 86, 64, 8
Contin et al. (2022)	2.01	26.5	0.95, 1.24, 6.20	$C_{50}$ : 54.1
Brendel et al. (2007)	1.46	28.2	1.35, 1.62	$C_{50}$ : 38.4
Mao et al. (2013)	1.80, 1.17, 1.55	63, 93.7, 7.3	0.81, 1.59, 0.60	$C_{50}$ : 2.5, 1.53, 2.1; $EC_{50}$ : 31.8
Westin et al. (2011)	1.37	NR	0.72, 17.9	$C_{50}$ : 1.41, 6.28; $EC_{50}$ : 4.26

NR, not reported. Values are listed as reported in the original studies. When both $C_{50}$ and $EC_{50}$ were available, both are reported for reference. For simulation and optimization analyses, a single potency definition was selected and applied consistently with unit harmonization.

### Comparison of dosing strategies

Parkinson’s disease exhibits substantial inter-individual heterogeneity, making individualized dosing an important consideration for optimizing therapeutic outcomes. Table 3 compares representative dosing scenarios across three illustrative virtual patient profiles examined within the simulated PK–PD framework: (i) early-stage disease with mild symptoms, (ii) moderate-to-advanced disease with pronounced motor fluctuations, and (iii) younger patients with higher physical activity levels. These profiles differ in age, body weight, disease stage, and therapeutic objectives, leading to distinct regimen requirements.

**TABLE 3:** Comparison of illustrative patient profiles and simulated dosing scenarios across representative Parkinson’s disease conditions. The table summarizes scenario-specific dose size, frequency, and timing explored within the proposed PK-PD framework. All values represent *in silico* selections for model exploration and are not prescriptive clinical recommendations.

Attribute	Case 1: Early-stage, mild symptoms	Case 2: Moderate-to-advanced disease with motor fluctuations	Case 3: Younger, high physical activity
Age	60 years	72 years	50 years
Weight	60 kg	75 kg	70 kg
Disease stage	Early stage	Moderate to advanced	Early to moderate
Key symptom context	Mild tremor, mild rigidity	Prominent tremor and rigidity; higher “OFF” burden	Symptoms worsen during exertion
Therapeutic target^†^	1.0–2.0 mg/L	2.5–4.0 mg/L	1.2–2.5 mg/L
Dosing interval	Every 8 h	Every 4 h	Every 8 h plus pre-activity dose
Dose explored^‡^	100–150 mg per dose	200–300 mg per dose	150 mg plus 50 mg pre-activity
$k_{a}$ *	2.0 $h^{-1}$	3.0 $h^{-1}$	4.0 $h^{-1}$
CL*	50 L/h	65 L/h	85 L/h
Expected simulated outcome	Stable mild exposure with lower dyskinesia risk	Potential reduction in simulated “OFF” periods	Potential improvement in symptom control during high activity

*denotes that scenario-specific simulation parameters were used for illustrative purposes and do not represent patient-fitted values.

^†^ Therapeutic target refers to simulated brain/effect-site concentration, not a clinical plasma threshold.

^‡^ Dose values indicate dose per administration, not total daily dose.

Scenario-specific simulation parameters were used for illustrative purposes and do not represent patient-fitted values.

For Case 1, smaller dose levels (100–150 mg per dose) administered at 8-h intervals were evaluated to assess whether symptom control could be explored while limiting excessive dopaminergic exposure. For Case 2, more frequent dosing schedules (e.g., every 4 h) with higher dose levels (200–300 mg per dose) were examined to investigate potential reductions in simulated OFF periods. For Case 3, an additional pre-activity dose was introduced to explore stabilization of motor response during periods of increased physical exertion.

Overall, these illustrative scenarios demonstrate how individualized PK–PD modeling can support comparative evaluation of alternative regimens across heterogeneous patient conditions and help identify dose adjustments aligned with distinct clinical priorities.

## Discussion

This study presents a quantitative PK–PD framework for exploring levodopa therapy in Parkinson’s disease and demonstrates how mechanistic modeling can link pharmacological processes with individualized dosing considerations. Using literature-informed parameters and simulation-based optimization, the proposed framework captures key features of levodopa exposure and therapeutic response across representative patient profiles. By explicitly accounting for variability in absorption, distribution, clearance, and pharmacodynamic sensitivity, the model provides a structured *in silico* environment for evaluating how alternative dosing schedules affect exposure stability, the likelihood of simulated “OFF” periods, and the potential for excessive dopaminergic stimulation associated with dyskinesia. These findings underscore the value of PK–PD modeling as a mechanistically interpretable framework for investigating treatment variability and informing future personalized levodopa dosing research.

### Summary of dosing comparisons

The illustrative dosing comparisons summarized in Table 3 highlight the flexibility of the proposed framework across heterogeneous clinical scenarios. For early-stage profiles with mild symptoms, lower dose levels administered at longer intervals were explored to assess whether stable exposure could be maintained while limiting overtreatment. In contrast, moderate-to-advanced profiles required more frequent dosing to examine strategies aimed at reducing simulated motor fluctuations. For younger and physically active profiles, incorporation of a pre-activity dose provided a mechanistic basis for exploring transient symptom worsening during exertion. Collectively, these scenarios emphasize that a single fixed dosing regimen is unlikely to be optimal across the diverse Parkinson’s disease population and that model-based personalization offers a systematic approach for comparing alternative dosing strategies.

### Comparison with previous studies

The findings are consistent with prior pharmacokinetic and PK–PD studies of levodopa. Earlier work by Troconiz et al. (Troconiz et al., 1998) and Chan et al. (2005) characterized levodopa exposure and response under predefined regimens but did not address individualized dosing exploration across heterogeneous patient profiles. The present framework extends these approaches by integrating parameter-driven simulations within a unified *in silico* setting. In addition, the results align with concepts discussed by Jorga et al. (2000) regarding the potential benefits of more continuous dopaminergic stimulation for reducing “OFF” episodes. In this context, the proposed model provides a quantitative platform to examine how algorithmic scheduling may promote more stable brain or effect-site exposure. The growing clinical interest in alternative delivery strategies further underscores the relevance of simulation-based evaluation of dosing approaches that may mitigate variability associated with oral administration. From a translational perspective, this relevance may be further strengthened by integration with digital biomarkers, wearable monitoring systems, and technology-assisted symptom assessment platforms capable of capturing response fluctuations in real-world settings (Schröter et al., 2022; Chan et al., 2005; Contin et al., 2022). In such a setting, computational PK–PD frameworks may serve as bridges between pharmacological exposure metrics and patient-specific symptom trajectories, thereby supporting future model-informed precision dosing research (Minichmayr et al., 2024; Kumar et al., 2018).

### Limitations and future perspectives

Several limitations warrant consideration. First, the framework relies on literature-derived parameters and simulated virtual cohorts; thus, the results should be interpreted as hypothesis-generating rather than as prescriptive clinical recommendations. Second, gastrointestinal dysfunction, such as delayed gastric emptying and variable intestinal absorption, was not modeled explicitly and may play a critical role in advanced disease. The present framework does not explicitly model food effects, COMT inhibitors, MAO-B inhibitors, or other co-medications, and these factors were therefore treated as omitted sources of variability in the current simulations. This is an important limitation because gastrointestinal barriers to levodopa transport and absorption have been increasingly recognized as key contributors to exposure variability and erratic therapeutic response in Parkinson’s disease (Leta et al., 2023; Nyholm, 2006). Future extensions should incorporate mechanistic gastrointestinal components, including transit-related delays or absorption heterogeneity, adjunct therapies such as COMT or MAO-B inhibitors, and additional patient-specific covariates. Among these planned extensions, incorporation of a mechanistic gastrointestinal sub-model to explicitly capture delayed gastric emptying and its impact on levodopa absorption dynamics will be prioritized in the next stage of model development. Validation against prospective clinical datasets and integration with wearable or digital monitoring tools could further enhance translational relevance.

## Conclusion

This study proposes a literature-informed PK–PD framework for exploring individualized levodopa dosing strategies in Parkinson’s disease. The model captures representative pharmacokinetic and pharmacodynamic behaviors across simulated patient profiles and provides a quantitative *in silico* platform for comparing dosing schedules under inter-patient variability. By integrating simulation-based optimization with sensitivity-informed parameter exploration, the framework enables systematic assessment of strategies aimed at maintaining therapeutic exposure while reducing patterns associated with motor “OFF” periods and excessive dopaminergic stimulation. Overall, the results support the potential value of model-guided personalization as a step toward precision dosing. Future work should focus on prospective clinical validation, explicit modeling of gastrointestinal and adjunct-therapy effects, and integration with wearable monitoring technologies and alternative delivery routes to enhance clinical relevance and translational impact.

### Study highlights

#### What is the current knowledge on the topic?

Levodopa remains the most effective therapy for motor symptoms in Parkinson’s disease, but its clinical use is complicated by substantial inter-individual variability in pharmacokinetics and pharmacodynamics. Quantitative PK–PD models have been developed, yet relatively few studies have provided a unified *in silico* framework for systematically comparing alternative dosing regimens.

#### What question did this study address?

This study developed a computational PK–PD model to simulate and compare levodopa dosing regimens across a virtual patient cohort with varying physiological and pharmacodynamic characteristics.

#### What does this study add to our knowledge?

The proposed framework integrates multi-compartment pharmacokinetics, effect-site dynamics, and inter-patient variability into a reproducible *in silico* platform for comparative dose evaluation.

#### How might this change drug discovery, development, and/or therapeutics?

This model-informed approach may support future precision dosing research and help guide optimization of levodopa therapy in Parkinson’s disease.

## Data Availability

The raw data supporting the conclusions of this article will be made available by the authors, without undue reservation.
